# Effects of Feeding Rumen-Protected Sunflower Seed and Meal Protein on Feed Intake, Diet Digestibility, Ruminal, Cecal Fermentation, and Growth Performance of Lambs

**DOI:** 10.3390/ani9070415

**Published:** 2019-07-04

**Authors:** Andrés Haro, Javier Gonzalez, Trinidad de Evan, Jesus de la Fuente, María Dolores Carro

**Affiliations:** 1 Departamento de Producción Agraria, ETSIAAB, Universidad Politécnica de Madrid, Ciudad Universitaria, 28040 Madrid, Spain; 2 Departamento de Producción Animal, Facultad de Veterinaria, Universidad Complutense de Madrid, 28040 Madrid, Spain

**Keywords:** protein protection, malic acid, heat, in vitro rumen fermentation, sunflower seed and meal, growing lambs

## Abstract

**Simple Summary:**

Due to inefficient use of nitrogen (N) in rumen, ruminants have a low efficiency of N utilization. A large part of ingested N is excreted into the environment without being used by animals. The objective of this study was to analyze the efficacy of a treatment combining malic acid and heating (MAH) to protect the protein in sunflower seeds (SS) and meal (SM) against rumen degradation and to improve the growth of lambs. Two high-cereal concentrates, either including untreated or MAH-treated SS and SM, were fed to two homogeneous groups of lambs. Further, feed intake, diet digestibility, and lamb growth from 14 to 26 kg body weight were assessed. An in vitro trial indicated that the MAH-treatment modified the rumen fermentation pattern of both SS and SM, and it reduced the NH_3_-N concentrations for SM. However, there were no significant effects of the MAH-treatment on feed intake, diet digestibility or growth of lambs. The hot carcasses of the lambs fed the MAH-treated concentrate were 7.9% heavier but not statistically different to those of the untreated group. In conclusion, under the conditions of the present study the MAH treatment did not improve the growth performance of lambs.

**Abstract:**

The objective of this study was to analyze the efficacy of a treatment (MAH) of sunflower seed (SS) and meal (SM) with a malic acid solution (1 M; 400 mL/kg) and heating (150 °C, 2 h) to protect protein against rumen degradation and to improve the growth of lambs. Two homogeneous groups of 12 Lacaune lambs each (14.2 ± 0.35 kg body weight) were fed either a concentrate including untreated SS and SM or a concentrate with MAH-treated SS and SM. Lambs were fed concentrate and barley straw ad libitum for 40 days (about 26 kg body weight); feed intake and growth of lambs were recorded; blood samples were taken on days 0, 20, and the slaughter day for analysis of urea-N and amino acid-N; diet digestibility was determined; and ruminal and cecal samples were collected after slaughter. The in vitro incubation of both concentrates with sheep ruminal fluid for 12 h showed that the MAH-treatment tended to reduce NH_3_-N concentrations and increased propionate production. However, there were no differences (*p* > 0.05) between groups in any of the tested variables in the in vivo trial.

## 1. Introduction

Ruminants usually have a lower efficiency of nitrogen (N) utilization than non-ruminant animals and a large part of the ingested N is excreted into the environment, thus contributing to both soil and water eutrophication. This problem is especially marked in productive ruminants with high protein requirements, such as growing and high-producing dairy animals. Rumen metabolism has been identified as the single most important factor contributing to the inefficient use of N in ruminants [1]. Nitrogen losses in the rumen are mainly due to an imbalance between protein degradation and the use of N for microbial protein synthesis, which results in high ruminal NH_3_-N concentrations [2]. In addition, recent studies [3,4] indicate that protein degradation also contributes to CH_4_ emissions, as a consequence of the fermentation of the carbon chains resulting from amino acid deamination. Therefore, reducing protein degradation in the rumen can not only increase the amount of feed proteins available for intestinal digestion and the amount of amino acids absorbed but also can reduce polluting emissions.

Several treatments such as heating and the treatment with acids have proven to be useful for protecting proteins from microbial degradation in the rumen, but the efficacy of these treatments varies with different factors, such as the characteristics and the dose of the acid used and the intensity and duration of the heat treatment [3]. Previous studies by our group [3,5,6] have shown the effectiveness of a combined malic acid and heat treatment to reduce sunflower protein degradation and to improve in vitro ruminal fermentation, but its efficacy has not yet been tested in vivo. Sunflower protein is widely used in ruminant nutrition because it is rich in sulfur-containing amino acids and tryptophan, but it is also extensively degraded in the rumen [3]. The objective of this research was therefore to analyze the effects of this treatment applied to sunflower meal (SM) and seed (SS) when both feeds were included in a high-cereal concentrate for growing lambs on productive performance, diet digestibility, and rumen and cecal fermentation. In addition, the in vitro ruminal fermentation of both untreated and treated SM and SS was assessed.

## 2. Materials and Methods

All procedures involving animals were conducted in accordance with the Spanish guidelines for experimental animal protection (Royal Decree 53/2013 1st February on the protection of animals used for experimentation or other scientific purposes). The experimental protocols were approved by the General Direction of Livestock and Agriculture of the Community of Madrid (Approval number PROEX 035/17).

### 2.1. Experimental Diets

Two experimental concentrates with the same feed ingredients excepting SS and SM, which were included either untreated (control concentrate) or treated with malic acid and heat (MAH concentrate) were formulated. Ingredient and chemical composition of both concentrates is shown in Table 1. The MAH treatment consisted in spraying successively 15 kg of SS (previously ground through 4 mm screen) and 18.3 kg of SM in a concrete mixer with a 1 M malic acid solution (400 mL/kg of feed) using a sprayer. Both fractions were then mixed, spread on stainless steel trays, and allowed to rest for 15 min at room temperature. The mixture was dried in a forced air oven at 150 °C for 2 h. Finally, the oven was turned off and the treated material was left in the oven to increase the thermal impact but avoiding oil exudation and burning. During the drying process, the feeds were manually stirred every 30 min during the 2 h of heating and every 60 min for the following 5 h. This process was repeated during three consecutive days to obtain the amount of SS and SM (45 and 55 kg, respectively) needed for preparing the MAH-concentrate. Untreated SS (ground through 4 mm screen) and SM were mixed homogeneously in this same proportion to prepare the control concentrate. During the MAH treatment, samples of SS and SM were individually taken for being tested in the in vitro trial. The two concentrates were formulated with the same ingredients to contain (DM basis) 155 g/kg of crude protein (CP) and 56 g/kg of ether extract, and both were pelleted (4 mm diameter). The CP supplied by SM represented about 35% of total CP, whereas the ether extract from SS was about 66% of total ether extract in both concentrates.

### 2.2. In Vitro Trial

Four adult rumen-fistulated sheep (67.8 kg ± 2.39 kg body weight) were used as rumen fluid donors for the in vitro incubations. Animals were individually housed in floor-pens and had free access to water and a mineral-vitamin block over the trial. Sheep were fed a mixed diet of grass hay and a commercial concentrate in 1:1 ratio at a daily rate of 45 g per kg of body weight^0.75^ administered in two equal meals. The diet contained 913, 150, 365, and 160 g of organic matter (OM), crude protein (CP), neutral detergent fiber (NDF), and acid detergent fiber (ADF) per kg, respectively.

Samples of SS and SM either untreated or treated and from both concentrates were ground through a 1 mm screen before analysis of chemical composition and in vitro fermentation with buffered rumen fluid. Two in vitro trials were conducted on different days using the same methodology and in each of them there were four replicates per feed sample by using the ruminal fluid from each sheep as inoculum (1 vial per feed and inoculum). The first in vitro trial was performed to assess the gas production kinetics of the samples, whereas the objective of the second trial was to determine the main fermentation parameters and CH_4_ production. Ruminal contents of each sheep were obtained before the morning feeding, strained through four layers of cheesecloth into thermal flasks, and immediately transported to the laboratory. The fluid of each sheep was independently mixed with a pre-warmed (39 °C) culture medium [7] in a proportion 1:4 under CO_2_ flushing. The medium of Goering and Van Soest [7] was modified to avoid N supply by replacing the (NH_4_)HCO_3_ with NaHCO_3_ and excluding the trypticase. Samples (200 mg of DM) of each feed were accurately weighed into 60 mL vials, which were filled up with 20 mL of the buffered rumen fluid using a peristaltic pump (Watson-Marlow 520UIP31; Watson-Marlow Fluid Technology Group, Cornwall, UK), sealed with rubber stoppers, and incubated at 39 °C for 120 h. Gas production was measured at 2, 4, 6, 9, 12, 16, 21, 25, 30, 35, 48, 60, 72, 96, and 120 h using a pressure transducer (Delta Ohm DTP704-2BGI, Herter Instruments SL, Barcelona, Spain) and a plastic syringe. The gas produced at each measurement time was released to prevent gas accumulation. Additional vials without substrate (blanks; two per inoculum) were included to correct the gas production values for the gas released from endogenous substrates.

The second in vitro trial lasted for 12 h. Gas production was measured and a gas sample (10 mL) was stored in an evacuated tube (Terumo Europe N.V., Leuven, Belgium) for analysis of CH_4_ concentration. Vials were uncapped, their content was homogenized, the pH was immediately measured (Crison Basic 20 pH-meter, Crisson Instruments, Barcelona, Spain), and 3 mL of vials content were mixed with 3 mL of 0.5 M HCl for analyses of volatile fatty acid (VFA) and NH_3_-N concentrations. Samples were frozen at −20 °C until analysis.

### 2.3. In Vivo Trial

Next, 24 male Lacaune lambs (14.2 ± 0.35 kg body weight) were distributed into two homogeneous groups according to their body weight; each group was randomly assigned to one of the two experimental concentrates (CON and MAH). Lambs were housed in individual pens (1 m × 1 m) with slatted floors, placed at 1 m above the floor, and equipped with two feeders and an automatic drinker. Pens were placed in a temperature-controlled room set at 20 °C. During the experiment (40 days), lambs were fed ad libitum concentrate and barley straw, and had free access to fresh water. Concentrate and straw intake was monitored twice per week and all lambs were weighed weekly. Samples of offered concentrate and straw were taken weekly for analysis of chemical composition.

After 30 days of trial, diet digestibility was measured in 10 lambs per treatment. Trays (1 m × 1 m) provided with holes were placed under the slatted floor of each pen for feces collection. Total feces voided by each lamb in 24 h were quantitatively collected for six days. An aliquot (10%) of total fecal output was collected each day for digestibility determination. Daily samples of feces were pooled to form a composite sample for each lamb and dried to constant weight before analysis.

On days 0, 20, and the slaughter day, blood samples were collected from each lamb via jugular venipuncture into tubes containing EDTA immediately before feeding. Samples were centrifuged (6000× *g* for 10 min at 4 °C), and the plasma was immediately frozen (−20 °C) until determination of urea-N and amino acid-N. Due to a problem during the storage of the samples taken on day 20, they could not be analyzed for urea-N, and values for this sampling are not reported.

After 39 days, lambs were weighed and slaughtered at a commercial slaughterhouse (Colmenar Viejo, Spain) on two different days (six lambs of each treatment per day). The carcass was weighed immediately after slaughter and weighed again after 24 h of airing in a cold chamber (4 °C). After removing the full gastrointestinal tract samples from rumen and cecal contents were immediately taken. The ruminal content was homogenized, a sample of about 300 g was filtered through four layers of gauze, and the pH of the fluid was immediately measured (Crison Basic 20 pH-meter, Crisson Instruments, Barcelona, Spain). Then, 2 mL of fluid were mixed with 2 mL of 0.5 N HCl. In addition, 2 g of cecal content were weighed and mixed with 2 mL of 0.5 N HCl. Samples were immediately frozen at −20 °C until analysis of NH_3_-N and VFA concentrations.

Finally, rumen papillae characteristics were evaluated as described by Carrasco et al. [8]. Briefly, a 10 × 10 cm rumen wall sample was collected from the ventral area of the rumen after rumen content sampling. The ventral area of the rumen was identified using the oesophagus and the spleen as physical references. Samples were washed with saline solution and the excess of solution was removed from the surface with tissue paper. Samples were then displayed on a white surface under an intense and homogeneous light to evaluate their colour by assigning to each of them a score from 1 (pale) to 5 (dark). The evaluation was performed by four trained persons, who were blind to treatment allocation, and the average score was used for statistical analysis. In addition, pictures of all samples were taken and downloaded into a computer. Six 1-cm^2^ areas were marked in each picture and the ruminal papillae within each area were counted. Finally, 10 papillae were randomly selected in each sample to measure their length and width using a Mitutoyo^®^ calibrator (Mitutoyo Corporation, Aurora, IL, USA) with a minimum resolution of 0.01 mm.

### 2.4. Chemical Analyses

Chemical composition of feeds, refusals, and feces was analyzed according the Association of Official Analytical Chemists [9] procedures for DM (ID 934.01), ash (ID 048.13), and ether extract (ID 945.16). Concentrations of NDF, ADF, and lignin were determined following the procedures of Van Soest et al. [10] and Robertson and Van Soest [11], respectively, using an ANKOM220 Fiber Analyzer unit (ANKOM Technology Corporation, Fairport, NY, USA). Sodium sulphite and α-amylase was used in the sequential analysis of NDF, ADF, and lignin, and ash-free values are reported. Nitrogen was measured by the Dumas combustion method employing a Leco FP258 N Analyzer (Leco Corporation, St. Joseph, MI, USA) and the amount of acid detergent insoluble N (ADIN) was determined by analyzing the N content in the residue obtained after the treatment of the sample with acid detergent solution. Concentrations of NH_3_-N and VFA in the rumen and cecal contents of lambs and in the vials content in the in vitro trial were determined by the phenol-hypochlorite method [12] and by gas chromatography [13], respectively. The analysis of CH_4_ was carried out following the procedure of Martínez et al. [14] using a gas chromatograph (Shimadzu GC 14B; Shimadzu Europa GmbH, Duisburg, Germany) equipped with a flame ionization detector and a column packed with Carboxen 1000 (Supelco, Madrid, Spain). All chemical analyses were performed in duplicate.

Plasma concentrations of urea-N were determined by the glutamate dehydrogenase enzymatic-spectrophotometric method of Gutman and Bergmeyer [15], and those of total amino acid-N by the 2,4-dinitrofluorobenzene (DNFB) method described by Goodwin [16].

### 2.5. Calculations and Statistical Analyses

Data of gas production in the in vitro trial were fitted with time using the exponential model: Gas = PGP (1 − e ^(− *c* (*t* − *lag*))^), where PGP is the asymptotic gas production, *c* is the fractional rate of gas production, *lag* is the time before starting gas production, and t is the time of gas measurement. Gas production parameters were estimated using the NLIN procedure of Statistical Analysis System [17] by an iterative least squares procedure. The average gas production rate (AGPR) was defined as the rate between the incubation start and the time at which half PGP is reached and it was calculated as AGPR = PGP *c*/[2 (ln2 + *c lag*)]. The amount of OM fermented (OMF) was calculated from acetate, propionate, and butyrate production in each vial as described by Demeyer [18].

In vitro data were analyzed independently for each feed (SS, SM, and concentrates) using the PROC MIXED of SAS [17] as a mixed model, in which the treatment was the fixed effect and the inoculum was considered as a random effect. In vivo data were analyzed as a one-way ANOVA using the GLM procedure of SAS [17]. Plasma metabolites were analyzed using the PROC MIXED of SAS [17] as a mixed model with repeated measures, in which the effects of the MAH treatment, sampling time, and the MAH treatment × sampling time interaction were fixed and animal was a random effect. Significance was declared at *p* < 0.05, whereas *p* < 0.10 values were considered as a trend.

## 3. Results and Discussion

There were only slight differences in chemical composition between both concentrates. Compared with CON, the MAH concentrate contained 10% less ether extract (Table 1) and more NDF (3.8%) and ADF (8.2%). The lower ether extract content was mainly due to the reduction in the ether extract content of SS produced by the MAH treatment (from 475 to 427 g/kg DM), which was attributed partly to a dilution effect associated with adding malic acid in the protective treatment and partly to fat losses during the heating process, as partial oil exudation was observed during this process. The slight increases in NDF and ADF may be associated with the heat treatment of sunflower seeds, as previously reported for different feeds [19,20].

### 3.1. In Vitro Trial

The effects of the MAH treatment on the in vitro fermentation of SS, SM, and both concentrates are shown in Table 2. Final pH ranged 6.28 to 6.77 and was not affected by MAH treatment for any substrate. Compared with the untreated SS, the MAH treatment increased PGP (*p* = 0.001) and AGPR (*p* = 0.043), which would indicate a stimulation of rumen fermentation. The greater VFA production and amount of OMF (*p* = 0.026 and 0.021, respectively) observed for MAH-treated SS are consistent with this idea and might be partly due to the direct fermentation of the added malic acid as malic acid is rapidly fermented by rumen microorganisms. In fact, Russell and Van Soest [21] reported that a greater concentration of malic acid (7.5 mM) than that used in the present study (4.5 mM) was fermented in vitro within the first 12 h of incubation. In contrast, for SM neither AGPR nor VFA production were affected by MAH treatment, although a trend (*p* = 0.058) to greater PGP was observed. The concentrate including MAH-treated SS and SM had greater AGPR (*p* = 0.020) and tended to have greater (*p* = 0.091) fractional rate of gas production than the CON concentrate, but VFA production was not affected. The variable effects of MAH treatment observed for the three substrates might be related to other additional effects of the treatment rather than malic acid fermentation itself. The increased fermentation observed in MAH-SS might be due to changes in their particle structure, which possibly improved microbial colonization. Thus, the partial oil exudation observed during the heat treatment may have reduced the potential toxic effects of free fatty acids on cellulolytic bacteria and consequently might have improved the microbial colonization of SS particles.

Vanegas et al. [3] applied the same protective treatment, with the exception that heating was applied for 1 h, to a different sample of SS and reported no effects on PGP, AGPR, and VFA production, but CH_4_ production and NH_3_-N concentrations were decreased. The lack of effect on CH_4_ production observed in our study might be due to the increased fermentation of SS due to MAH treatment, as CH_4_/VFA ratio was similar for both untreated and treated SS (2.27 and 2.35 mL/mmol, respectively). Concentrations of NH_3_-N were decreased by 5.5%, but differences did not reach the significance level; however, a protein protection could not be excluded, as this could have been compensated with the increased fermentation observed for MAH-treated SS. Differences in the results of Vanegas et al. [3] and in the present study may be associated with the increased heating in the present study, not only due to the greater time of heating but also to the greater humidity in the oven due to the larger quantity of SS treated (about 47 kg in the present study and 500 g in the study of Vanegas et al. [3].

The trend to greater PGP (*p* = 0.058) and the lack of effect on both VFA production and OMF observed when SM was MAH-treated are in good agreement with the results reported by Vanegas et al. [3] for the same feed. The protective effect of MAH treatment against protein degradation indicated by these authors was supported by the reduced (*p* = 0.009) NH_3_-N concentrations observed in our study for the MAH-treated SM compared with the untreated SM. The inclusion of MAH-treated SS and SM in the concentrate resulted in greater (*p* = 0.020) AGPR and a trend to lower (*p* = 0.065) NH_3_-N concentrations compared with CON concentrate. In addition, the lack of effects on VFA production and OMF indicates that MAH treatment had no detrimental effect on ruminal fermentation.

The MAH treatment affected VFA profile of the three substrates (SS, SM, and concentrate) and both an increase (*p* ≤ 0.044) in molar proportions of propionate and a decrease (*p* ≤ 0.023) in acetate/propionate ratio was observed for all of them. In addition, a reduction (*p* ≤ 0.015) in acetate molar proportions was observed for MAH-treated SS and SM (*p* = 0.003 and 0.015, respectively). The effects of malic acid or malate salts on VFA profile have been reported to vary with the incubated substrate [22,23], but an increase in propionate production has been observed in most in vitro studies because malate is an intermediate metabolite of the succinate pathway of propionate production by ruminal microorganisms [23]. Similar changes in VFA profile were observed by Vanegas et al. [3] for both SS and SM.

### 3.2. In Vivo Trial

As shown in Table 3, there were no differences between groups in concentrate, barley straw, and total feed intake, showing that the MAH treatment, which included malic acid at 1.06% of concentrate, did not negatively affect concentrate palatability. This agrees with previously reported results in growing lambs [24,25] and beef cattle [8,26], indicating that no negative effects of malic acid or malate salts on feed intake should be expected when they are included in the diet at levels up to 2.6%. Although lambs were fed barley straw ad libitum, the intake of barley straw was very low, ranging from 2.1 to 5.2% of total DM intake. This low intake of straw is consistent with that observed in previous studies in lambs under similar feeding conditions [24,27].

The MAH treatment did not affect final body weight of lambs, average daily gain, feed conversion rate, and carcass traits (Table 3), which is in agreement with the lack of differences observed by Díaz-Royón et al. [28] when applying the MAH-treatment to SM and spring pea protein. The present study and that of Díaz-Royón et al. [28] used similar feeding systems (high-cereal concentrates and cereal straw fed ad libitum), lambs had similar initial and final weights, and the amount of malic acid included in the MAH-treated concentrates was also comparable (1.1%) but differed in the MAH-treated protein feeds included in the concentrate.

Organic matter digestibility tended to be greater (*p* = 0.074) in MAH-fed lambs than in the control group, but there were no differences in the digestibility of CP, NDF, and ADF. Most studies have shown a lack of effect of malic acid or malate salts supplementation, as feed additives on diet digestibility in lambs [24] and beef cattle [26], although Flores et al. [29] observed an increase in diet digestibility by supplementing Manchega and Lacaune fattening lambs with disodium–calcium malate at 2 and 4 g/kg DM and Mungói et al. [25] observed a reduction of diet digestibility by using the same product at 1 g/kg DM in the diet of Manchega fattening lambs. These discrepancies in the results may be related to differences in the composition of the concentrate used in the different trials. In addition, it should be considered that in the present study malic acid was not used as feed additive and the heat treatment might have modified its effect on rumen fermentation.

Blood concentrations of urea-N are markedly influenced by the amount of NH_3_-N absorbed from the rumen and therefore they reflect the balance between protein degradation and the use of NH_3_-N for microbial protein synthesis. However, urea-N concentrations also reflect the amount of urea produced by the liver from amino acid catabolism [2]. As shown in Table 4, plasma concentrations of urea-N and amino acid-N were not affected by MAH treatment. Urea-N concentrations were steady over the trial, but amino-N decreased (*p* < 0.001) with advancing time. Eryavuz et al. [30] also observed that plasma concentrations of amino-N in growing lambs decreased with age, which was attributed to the fact that protein requirements decrease with age and therefore the synthesis of amino acids in the liver is reduced.

The lack of effects of MAH-treatment on post-mortem VFA concentrations and VFA profile in the rumen and the cecum (Table 5) is in agreement with the results of Carro et al. [24], who observed that supplementation of the concentrate fed to growing lambs with 0.4 or 0.8% of malate salts did not affect ruminal pH, total VFA concentrations or VFA profile measured after slaughtering. Similar results have been reported in beef cattle [8,26] and dairy cows [31] by supplementing malic acid or malate salts up to 2.64% of total diet. As discussed by Carro et al. [24], it is possible that greater levels of malate would be necessary to detect significant effects on in vivo VFA production and diet degradability. Moreover, it has to be considered that, when determining VFA concentrations in the rumen, VFA proportions may be unrepresentative of net production [32].

Ruminal papillae characteristics were in the range reported by others for growing lambs fed high-cereal concentrates [33,34] and were not affected by MAH treatment (Table 5). However, the color of the ruminal epithelium was darker (*p* = 0.003) in the lambs fed the MAH-concentrate compared with the control group, which might be due either to a corrosive action of malic acid or to a greater abrasion of the MAH-treated sunflower husks. However, the lack of differences between groups in any other variable tested would indicate that ruminal absorption was not negatively affected by the MAH-treatment.

In summary, the MAH treatment was effective in increasing the in vitro fermentation of SS, reducing the in vitro protein degradability of SM, and modifying the VFA profile towards greater propionate production. However, under the conditions of the present study the inclusion of MAH-treated SS and SM in a concentrate for growing lambs did not influenced feed intake growth, diet digestibility or growth performance.

## Figures and Tables

**Table 1:** Ingredient and chemical composition of the experimental concentrates and barley straw. MAH: malic acid and heating.

Item	Concentrate ^1^		Barley Straw
	Control	MAH	
Ingredient (g/kg fresh matter)			
Untreated sunflower meal	109	-	
Treated sunflower meal	-	109	
Untreated sunflower seed	89.0	-	
Treated sunflower seed	-	89.0	
Soybean meal	50.0	50.0	
Wheat	196	196	
Barley	264	264	
Corn	263	263	
CO_3_Ca	22.4	22.4	
NaCl	4.8	4.8	
Mineral-vitamin premix	2.0	2.0	
Chemical Composition			
Dry matter (DM; g/kg fresh matter)	899	895	923
Organic matter (g/kg DM)	941	940	891
Crude protein (g/kg DM)	156	153	29.0
Ether extract (g/kg DM)	56.1	50.5	16.0
Neutral detergent fiber (g/kg DM)	184	191	719
Acid detergent fiber (g/kg DM)	67.1	72.6	380
Lignin (g/kg DM)	10.5	11.2	92.1

^1^ Both sunflower meal and seeds were treated with malic acid and heat for protein protection in the MAH treatment.

**Table 2:** Gas production parameters and in vitro fermentation parameters of sunflower seed, sunflower meal and concentrates containing both sunflower products either untreated (control) or treated with malic acid and heat (MAH) for protecting protein against ruminal degradation ^1^.

Item	Sunflower Seed (SS)				Sunflower Meal (SM)				Concentrate			
	Control	MAH	SEM ^2^	*p* =	Control	MAH	SEM^2^	*p* =	Control	MAH	SEM ^2^	*p* =
**Gas production parameters** **^3^**												
PGP (mL/g dry matter)	63.3	82.1	1.09	0.001	147	164	3.94	0.058	270	266	3.93	0.466
*c* (%/h)	4.40	4.56	0.52	0.937	5.48	4.98	0.17	0.117	5.60	5.88	0.08	0.091
*Lag* (h)	0.90	0.28	0.27	0.197	1.11	0.48	0.25	0.171	3.20	2.98	0.09	0.167
AGPR (mL/g dry matter)	1.79	2.52	0.15	0.043	5.36	5.69	0.26	0.430	8.57	8.83	0.04	0.020
**Ruminal parameters**												
CH_4_ (mL)	1.69	1.96	0.092	0.100	3.84	4.07	0.359	0.424	5.59	5.95	0.221	0.330
NH_3_-N (mg/L)	211	207	2.8	0.125	322	279	4.99	0.009	161	146	3.71	0.065
Total Volatile fatty acids (VFA; mM)	37.2	41.7	0.77	0.026	55.2	56.0	2.51	0.822	63.6	67.4	1.96	0.265
Individual VFA (mol/100 mL)												
Acetate	67.7	65.5	0.18	0.003	66.3	63.6	0.38	0.015	62.8	61.5	0.40	0.349
Propionate	20.3	23.0	0.32	0.010	22.0	25.7	0.38	0.006	23.2	24.4	0.26	0.044
Butyrate	8.17	7.54	0.22	0.141	7.63	7.12	0.11	0.044	11.9	11.5	0.52	0.616
Minor VFA	3.87	4.08	0.111	0.292	4.13	3.63	0.178	0.143	2.89	2.68	0.235	0.585
Acetate/propionate (mol/mol)	3.37	2.86	0.071	0.015	3.03	2.49	0.060	0.006	2.70	2.55	0.022	0.023
OMF (g/kg)	217	241	39.2	0.021	331	337	13.7	0.805	397	419	6.9	0.200

^1^ Substrates (200 mg dry matter) were incubated with sheep ruminal fluid for 120 h for measuring gas production kinetics and for 12 h for measuring fermentation parameters (n = 4). Control concentrate contained untreated SS and SM and MAH concentrate contained MAH-treated SS and SM. ^2^ Standard error of the mean. ^3^ PGP: potential gas production; *c*: fractional rate of gas production; *lag*: time before the onset of gas production; AGPR: average gas production rate until half of PGP is reached; OMF: organic matter fermented estimated from VFA production as described by Demeyer [18]; minor VFA: calculated as the sum of isobutyrate, isovalerate, and valerate proportions.

**Table 3:** Initial and final body weight, feed intake, average daily gain, feed conversion rate, carcass traits, and diet digestibility of growing lambs fed barley straw and concentrate containing sunflower meal and seed either untreated (control) or treated with malic acid and heat (MAH).

Item	Concentrate		SEM ^1^	*p =*
	Control	MAH		
Initial body weight (kg)	14.2	14.2	0.31	0.975
Final body weight (kg)	26.5	27.1	0.46	0.357
Feed intake (g/d)				
Concentrate	873	915	21.6	0.184
Straw	31.3	32.9	2.15	0.613
Total	904	948	2.1	0.178
Average daily gain (g/d)	314	329	14.8	0.473
Feed conversion rate (g/g)	2.98	2.92	0.161	0.766
Carcass traits				
Hot carcass weight (kg)	12.7	13.7	0.21	0.212
Cold carcass weight (kg)	12.4	12.8	0.21	0.198
Cold carcass yield (%)	46.8	47.2	0.88	0.736
Diet digestibility (g/kg)				
Organic matter	807	815	7.0	0.074
Crude protein	719	731	9.9	0.394
Neutral detergent fiber	506	505	19.9	0.962
Acid detergent fiber	393	383	21.7	0.335

^1^ Standard error of the mean.

**Table 4:** Plasma concentrations of urea-N and amino acid-N in lambs fed barley straw and concentrate containing sunflower meal and seed either untreated (control) or treated with malic acid and heat (MAH) at the start (0), middle (day 20), and end of the trial.

Item	Treatment (TR)	Sampling Day			SEM_TR_^1^	SEM_T_^1^	*p* =		
		0	20	Slaughter Time			TR	Time	TR × Time
Urea-N [mg/100 mL]	Control	28.5	-	28.3	1.13	1.13	0.755	0.677	0.795
	MAH	28.4	-	27.3					
Amino acid-N [mg/100 mL]	Control	0.74 ^b^	0.66 ^b^	0.33 ^a^	0.060	0.073	0.500	<0.001	0.383
	MAH	0.87 ^b^	0.60 ^a^	0.34 ^a^					

^a,b^ Within each parameter and row, different superscripts indicate differences among sampling times (*p* < 0.05; LSD test). ^1^ SEM_TR_: standard error of the mean for treatment effect; SEM_Time_: standard error of the mean for time effect.

**Table 5:** Post-mortem fermentative parameters in the rumen and cecum of lambs fed barley straw and concentrate containing sunflower meal and seed either untreated (control) or treated with malic acid and heat (MAH).

Item	Concentrate		SEM ^1^	*p* =
	Control	MAH		
Rumen				
pH	5.17	5.26	0.060	0.510
Total VFA (mM)	152	156	1.8	0.778
Individual VFA (mol/100 mol)				
Acetate	49.0	48.5	0.59	0.732
Propionate	41.0	41.6	0.61	0.757
Butyrate	5.87	5.98	0.48	0.919
Isobutyrate	0.48	0.48	0.139	0.931
Isovalerate	0.52	0.49	0.143	0.831
Valerate	2.52	2.49	0.251	0.915
Caproate	0.56	0.53	0.172	0.878
Acetate/propionate (mol/mol)	1.21	1.19	0.141	0.852
NH_3_-N (mg/L)	51.8	52.1	2.11	0.989
Rumen wall characteristics				
Colour ^2^	1.71	2.78	0.267	0.003
Papillae length (mm)	3.94	4.01	0.157	0.779
Papillae numbers (per cm^2^)	92.2	94.9	4.03	0.639
Cecum				
Total VFA (mM)	172	171	1.6	0.910
Individual VFA (mol/100 mol)				
Acetate	66.4	64.1	0.69	0.324
Propionate	19.4	20.1	0.61	0.647
Butyrate	11.5	13.2	0.50	0.172
Isobutyrate	0.36	0.37	0.117	0.922
Isovalerate	0.30	0.29	0.109	0.877
Valerate	1.40	1.34	0.203	0.776
Caproate	0.69	0.67	0.203	0.919
Acetate/propionate (mol/mol)	3.61	3.29	0.262	0.360
NH_3_-N (mg/L)	76.6	63.1	1.97	0.485

^1^ Standard error of the mean. ^2^ Color was scored from 1 (pale) to 5 (dark).
